# Origin and Objects of the Epidemiological Society

**Published:** 1855-12

**Authors:** 


### TRANSACTIONS

OF THE

### EPIDEMIOLOGICAL SOCIETY OF LONDON.

---

### ORIGIN AND OBJECTS OF THE EPIDEMIOLOGICAL SOCIETY.

The Epidemiological Society for the investigation of epidemic diseases was instituted on the 30th July, 1850, at a meeting held at the Hanover Square Rooms, under the presidency of Lord Ashley, now the Earl of Shaftesbury.

The records of the Registrar-General had brought to light most important and astounding facts, regarding the great mortality from zymotic diseases, and the constant and active prevalence of the ordinary epidemics of this country,—as measles, scarlatina, hooping-cough, and typhus-fever.

Cholera, after an absence of sixteen years, an absence which had inspired a hope of its having left us for ever, had again visited our shores, and, during the two preceding years, had carried off between 50,000 and 60,000 of the population.

The rapid transit from the West Indies to this country, by means of steam, had brought us within a voyage of twelve days, instead of one of six weeks, from the domain of yellow-fever. And the arrival of vessels on our coasts, with yellow-fever actually on board, had taught us that the great scourge of the tropics is not always confined to certain degrees of latitude and conditions of temperature.

In Brazil, reckoned one of the healthiest climates of the world, yellow-fever, which had been unknown there for more than a century, was committing fearful ravages among all classes.

These and other considerations seemed to indicate, that the establishment of a Society, devoted to the investigation of past, as well as of existing epidemics, was in every way desirable, if indeed it was not imperatively demanded ; and the time also appeared peculiarly favourable for carrying out the inquiries contemplated by such a Society. It was well remarked by Dr. Babington, the president, in his address, on the opening of the first session of the Society,—"The peace of Europe, which it is to be wished no national jealousies may disturb, is very favourable to the interchange of scientific information among medical men of different countries ; and the facilities of communication, which the powers of steam have created, enable us to carry on, almost simultaneously, observations at different places remotely distant from each other. There probably never was a period in the world's history, when human knowledge was so advanced in all its branches as at the present time."

Having thus referred to the circumstances that led to the formation of the Epidemiological Society, it is presumed that a somewhat more detailed statement of the ends which the Society seeks to attain, as well as a brief account of what it has already done, and of its prosperity, may be acceptable.

In the "Objects of the Society," published in 1850, this Society purpose, among other things—

"To institute a rigid examination into the causes and conditions which influence the origin, propagation, mitigation, and prevention of epidemic diseases.

"To institute, in a spirit of careful inquiry, original and comprehensive researches into the nature and laws of diseases connected with soil and climate, of which ague, remittent and yellow fever, offer good example; of epidemics, such as typhoid-fever, prevailing in towns, villages, or rural districts ; in public institutions, as workhouses, prisons, lunatic asylums, and hospitals; in ships, barracks, and encampments : of epidemics like cholera, appearing in a locality, and spreading thence to distant countries: of the eruptive fevers, as scarlatina.

"In order to throw light upon the whole question of epidemic diseases, and as an object in itself of important inquiry, it will be within the scope of the Society to investigate the diseases prevailing extensively among domestic and other animals, as well as those that effect the vegetable kingdom.

"Immediately on the outbreak of an epidemic in any place, the Society will consider it its special province to institute a searching inquiry, tracing with scrupulous minuteness the history of the first cases.

"In the investigation of epidemic diseases the aim will be, to trace them up to their causes; to examine such causes thoroughly; and to ascertain whether they admit of prevention, and if so, by what means.

"The spread of epidemics will form another subject of inquiry; and, under this head, the influence of isolation and of unrestrained intercourse will be particularly investigated.

"It will be a further part of the Society's province to ascertain the operation of existing legal enactments which bear upon epidemic diseases: such as those which relate to quarantine; to vaccination; to the sale of unwholesome or adulterated food; and to the removal of nuisances: to inquire into the defects of these enactments, and point out such alterations as may be necessary for the protection of the public health. The Society also propose to communicate with the government and legislature on matters connected with the prevention of epidemic diseases.

"The Society will solicit the active and organised co-operation of government boards, medical corporate bodies, public institutions, and medical societies, and will invite the formation of co-operating societies, abroad and in this country, grafting the latter, when practicable, on existing medical societies.

"Lastly, it is their intention to receive, to read at their meetings, and to publish original papers and communications; to issue queries; to publish reports; to form statistical tables; to prepare illustrative maps; and to collect works relating to epidemic diseases."

Acting in conformity with the spirit and intention of the Society, as expressed in the above extracts from its objects, the council have put themselves in communication with the heads of various departments of the government at home; with many of the leading scientific men on the Continent, as well as in this country; and with high medical and other authorities in various parts of the globe, including India, America, and most of our own, as well as some foreign Colonial possessions

The following committees have been for some time formed. In some of them, the members are at present diligently employed in carrying out the investigations intrusted to them, but the operations of others, we regret to say, have been retarded from the want of pecuniary support.

The Committee on Small-pox and Vaccination;
The Cholera Committee;
The Epizootic Committee;
The Hospitals' Committee;
The Continued Fever Committee;
The Committee appointed to inquire into the Diseases affecting the Vegetable Kingdom.

Another most important committee was established in May last, to take into consideration the question of supplying the labouring classes with nurses in epidemic and other diseases. The progress of this committee will be seen in the Fifth Annual Report of the Society, which appears in another part of our pages.

The Small-pox and Vaccination Committee, about two years back, presented to the Council a report "On the State of Small-pox and Vaccination in England and Wales, and on compulsory Vaccination." Copies of this report were forwarded by order of the Council to the Secretary of State for the Home Department, and to Lord Lyttelton, for their assistance in the consideration and amendment of a measure for making vaccination compulsory, which had lately been introduced into the House of Lords by Lord Lyttelton, under the title of the "Vaccination Extension Act."

This report was considered to be of such importance, that it was printed by order of both Houses of Parliament; and the very considerable amendments that the vaccination bill underwent in its various stages were entirely due to suggestions emanating from the Committee, and contained in this report, and in a second or supplementary report on the same subject.

The Small-pox and Vaccination Committee have recently presented to the Council a very able and elaborate memoir, which has been forwarded by the president to Sir Benjamin Hall, President of the Board of Health. The main object of this memoir is to place the vaccinations in the United Kingdom under competent medical superintendence, and thus to insure their efficient performance, and a corresponding protection of public health.

It remains only to refer to the papers read at the monthly meetings of the Society. These communications, both as learned and as scientific records, are of the utmost value. Through the liberal patronage of the weekly medical press, they have been freely circulated, in the form of abstracts, throughout the world. From henceforth they will be published *in extenso* in these Transactions of the Society.

---

